# Vitamin D (1,25-(OH)_2_D_3_) Improves Endothelial Progenitor Cells Function via Enhanced NO Secretion in Systemic Lupus Erythematosus

**DOI:** 10.1155/2020/6802562

**Published:** 2020-10-16

**Authors:** Zhenhua Huang, Lixiang Liu, Shufen Huang, Jianbo Li, Shaozhen Feng, Naya Huang, Zhen Ai, Weiqing Long, Lanping Jiang

**Affiliations:** ^1^The Division of Emergency Medicine, The First Affiliated Hospital of Sun Yat-sen University, Guangzhou, China; ^2^Department of Gynecology, Seventh Affiliated Hospital of Sun Yat-sen University, Shenzhen, China; ^3^Department of Nephrology, The First Affiliated Hospital, Sun Yat-sen University, Guangzhou 510080, China; ^4^Key Laboratory of Nephrology, National Health Commission and Guangdong Province, Guangzhou 510080, China; ^5^Department of Clinical Laboratory, The First Affiliated Hospital, Sun Yat-sen University, Guangzhou, China

## Abstract

It has been proven that vitamin D was decreased and function of circulating endothelial progenitor cells (EPCs) was injured in systemic lupus erythematosus (SLE) patients. However, the effect of vitamin D on the function of EPCs *in vitro* and its mechanism need further study. Therefore, we investigated whether vitamin D improved the function of EPCs *in vitro*. The peripheral blood mononuclear cells of the participants were isolated from SLE patients and control subjects and cultured to EPCs. After the EPCs were treated with vitamin D (1,25-(OH)_2_D_3_), we evaluated the number, migratory and proliferative activities, and nitric oxide (NO) production of EPCs *in vitro* and detected vascular endothelial function by flow-mediated dilatation (FMD). We found that vitamin D in a dose-dependent manner improved number and migratory and proliferative activities of EPCs from SLE patients. Additionally, vitamin D upregulated NO production from EPCs *in vitro*. A significant correlation between the FMD and plasma NO level was found. There was also a correlation between number, migration, and proliferation of EPCs and NO production. Thus, the present findings indicated that vitamin D improved the function of EPCs from SLE patients via NO secretion.

## 1. Introduction

Systemic lupus erythematosus (SLE) is a prototype of autoimmune disease, which mainly affects young women. SLE has a high disability and mortality rate, which caused a heavy burden to families and society [1]. With the progression of diagnosis and treatment, the survival time of SLE patients has been greatly improved in recent years. However, the mortality and disability rates of atherosclerosis are gradually increasing [2]. In SLE patients, atherosclerosis occurs early and severely. Even after removing the impact of traditional risk factors, the relative risk of coronary heart disease in lupus patients is still as high as 8–10 times [3]. So far, the mechanism of atherosclerosis induced by systemic lupus erythematosus remains unclear.

Studies have shown that vascular endothelial structure and function damage was the initiating mechanism of atherosclerosis in SLE [4, 5]. The essence of vascular dysfunction is the imbalance between vascular injury and vascular repair, which is an important factor leading to target organ damage, such as the heart, brain, and kidney damage, and cardiovascular events [6, 7]. Therefore, the repair of vascular endothelial injury is an important measure to effectively prevent and treat SLE atherosclerosis and its complications. In recent years, endothelial progenitor cells (EPCs) have been found to be precursors of vascular endothelial cells, which can effectively repair vascular endothelial injury [8–10]. When the vascular endothelium is damaged, EPCs are released from the bone marrow to peripheral circulation passively, and by chemotaxis, adhesion, migration, and proliferation, EPCs accelerate vascular reendothelialization, which plays a key role in the process of vascular endothelial injury repair [10, 11]. A large number of studies have confirmed that cardiovascular risk factors can lead to different degrees of vascular endothelial injury, the number and function of EPCs decreased, and endothelial function also decreased accordingly, which indicates that this endothelial dysfunction is closely related to the number and function of EPCs [12].

Brachial artery endothelium-dependent vasodilation, as detected by flow-mediated dilatation (FMD), is a noninvasive and repeatable new technique for detecting vascular function, which can accurately reflect cardiovascular endothelial dysfunction [13]. A large number of studies have pointed out that FMD and EPCs are closely related in number and function, which indicates that EPCs are cell biological indicators that reflect the changes of vascular endothelial function [14]. The main physiological function of vitamin D is to regulate calcium and bone metabolism. Vitamin D deficiency is very common in SLE patients, and it can affect the function of vascular endothelium and lead to the occurrence and development of atherosclerosis [5, 15–17]. Therefore, what is the effect of vitamin D on endothelial progenitor cells in SLE patients? The relevant mechanism needs further study.

Nitric oxide (NO) is an endothelial relaxing factor and a highly active free radical. Previous studies have found that NO plays an important role in regulating endothelial progenitor cell adhesion, migration, and proliferation and mediating vascular repair as well [8, 14, 18]. Therefore, we hypothesized that the decrease of EPCs in SLE patients is related to the low level of active vitamin D, which may be regulated by NO. By observing the effect of vitamin D on EPCs and NO level in SLE patients, this study elucidated the underlying causes of atherosclerosis in SLE patients, thus laying a foundation for the clinical prevention and treatment of cardiovascular diseases in SLE patients and the discovery of new drug targets.

## 2. Method

### 2.1. Subject Recruitment

20 SLE patients and 20 age- and gender-matched control subjects were enrolled from the First Affiliated Hospital of Sun Yat-sen University in Guangzhou, China. The control subjects had no hypertension, heart disease, diabetes, or stroke history. The peripheral blood samples of another 40 SLE patients were collected and cultured to EPCs for 1,25-(OH)_2_D_3_ stimulation experiments. The protocol of this study was approved by the Ethics Committee of the First Affiliated Hospital of Sun Yat-sen University. The general clinical characteristics of subjects are listed in Table 1. Serum 25(OH)D was measured by highly sensitive enzyme-linked immunosorbent assay (ELISA, R&D Systems, Germany) according to the manufacturer's instructions.

### 2.2. Measurement of Flow-Mediated Dilation (FMD)

As previously described [19, 20], the brachial artery FMD was measured by a 5–12 MHz linear transducer on an HDI 5000 system (Philips Healthcare, the US).

### 2.3. Measurement of NO, VEGF, and GM-CSF Level Secreted by EPCs

NO, vascular endothelial growth factor (VEGF), and granulocyte-macrophage colony-stimulating factor (GM-CSF) levels in plasma or secretion by EPCs were evaluated as previously described [14]. We used ELISA kits to determine NO, VEGF, and GM-CSF levels according to the manufacturer's instructions.

### 2.4. Flow Cytometry and Cell Culture Assay to Assess the Number of Circulating EPCs

As previously described [14], the peripheral blood mononuclear cells of the participants were isolated using Ficoll density gradient centrifugation, following 7 days of culture. EPCs were incubated with 1,1′-dioctadecyl-3,3,3′,3′-tetramethylindocarbocyanine perchlorate-labeled acetylated LDL (DiI-acLDL, Thermo Fisher Scientific, the US) and incubated with fluorescein isothiocyanate- (FITC-) labeled lectin (Sigma-Aldrich, Germany). The samples were observed by two independent researchers using a phase-contrast fluorescence microscope (magnification, ×200). Cells demonstrating double-positive fluorescence were identified as differentiating EPCs. The EPCs were counted using a kinase-insert domain receptor (KDR; 4A Biotech, China) and antihuman CD34 (4A Biotech, China). The ratio of CD34 + KDR + cells per 100 peripheral blood mononuclear cells was the number of circulating EPCs.

### 2.5. Migration and Proliferation Assay of EPCs

EPC migration and proliferation assays were described in a previous study [14]. Migration was analyzed using a modified Boyden chamber. Briefly, 2 × 10^4^ EPCs, resuspended in 250 *μ*l EBM-2, were pipetted in the upper chamber of a modified Boyden chamber. Cell nuclei were stained with 4′,6-diamidino-2-phenylindole (DAPI). The proliferation of EPCs was analyzed as follows: EPCs were supplemented with 10 *μ*l MTT (Fluka, the US) and measured by optical density at 490 nm.

### 2.6. Treatment of Circulating EPCs by 1,25-(OH)_2_D_3_

Forty SLE patients' blood samples were divided into four groups randomly. The peripheral blood mononuclear cells were cultured to EPCs, which were digested by 0.25% trypsin to form a single cell suspension. Then, each group cells were treated with 1,25-(OH)_2_D_3_ (0, 1, 10, 50 nM, Sigma-Aldrich, the US) for 72 h, respectively.

### 2.7. Statistical Analysis

Normally distributed variables were expressed as mean ± SD and compared using unpaired *t* tests between two groups. Correlation coefficients were analyzed using Pearson's correlation. Differences were considered significant when *p* < 0.05. All statistical analyses were performed with SPSS statistical software 21.0 (SPSS, Inc., the US).

## 3. Result

### 3.1. Baseline Characteristics

As listed in Table 1, no significant differences were observed in age, body mass index (BMI), aspartate aminotransferase (AST), alanine transaminase (ALT), blood urea nitrogen (BUN), serum creatinine (Cr), and lipids level between SLE patients and, while the level of serum 1,25-(OH)_2_D_3_ in the SLE group was apparently lower than that of the control group (*p* < 0.05).

**Figure 1:**
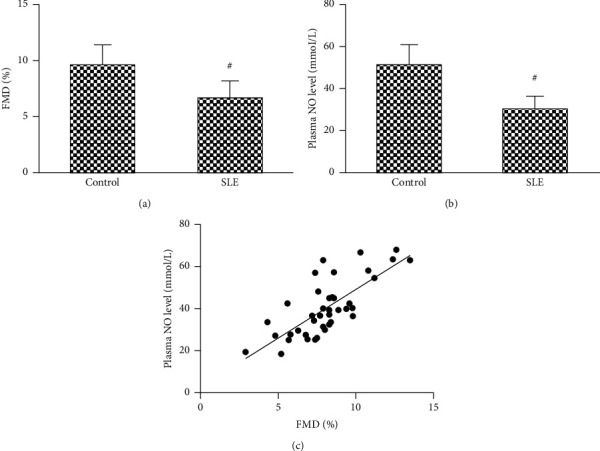
The FMD and plasma NO level in the two groups. The brachial artery FMD (a) and plasma NO level (b) in SLE patients were lower than that in the control subjects. FMD was positively correlated to plasma NO level (c). ^#^*P*<0.05 vs. control subjects.

As shown in Figure 1, compared with the healthy control subjects, the FMD and plasma NO level of SLE patients group are significantly decreased (Figures 1(a) and 1(b)). We also found that the FMD was correlated with the NO level, indicating that endothelium damage may be related NO decrease in SLE patients (Figure 1(c)).

**Figure 2:**
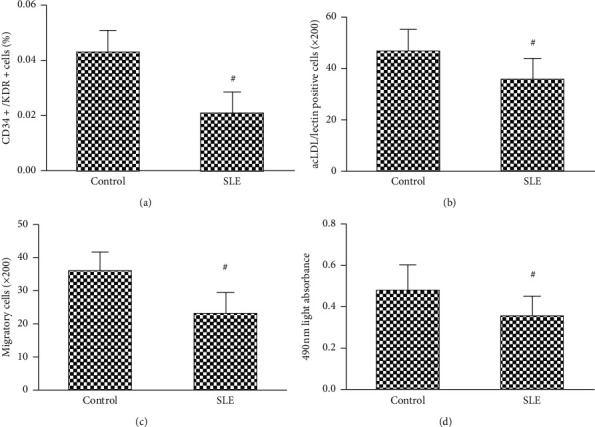
The number of circulating EPCs in the SLE patients and healthy controls. Evaluated by (a) FACS analysis and (b) phase-contrast fluorescent microscope, the number of circulating EPCs in SLE patients was lower than those in the control subjects. The migratory (c) and proliferative (d) activities of circulating EPCs in SLE patients were lower than those in the healthy control. Data are given as mean ± SD. ^#^*P*<0.05 vs. control subjects.

As shown in Figure 2, the number of EPCs was significantly decreased in the peripheral blood mononuclear cells from SLE patients, compared with control subjects (Figure 2(a)). Consistently, the migration and proliferation activities of EPCs from SLE patients were notably decreased compared to those from control subjects (Figures 2(b) and 2(c)).

### 3.2. The Effect of 1,25-(OH)_2_D_3_ on the Number, Migration, and Proliferation of EPCs

**Figure 3:**
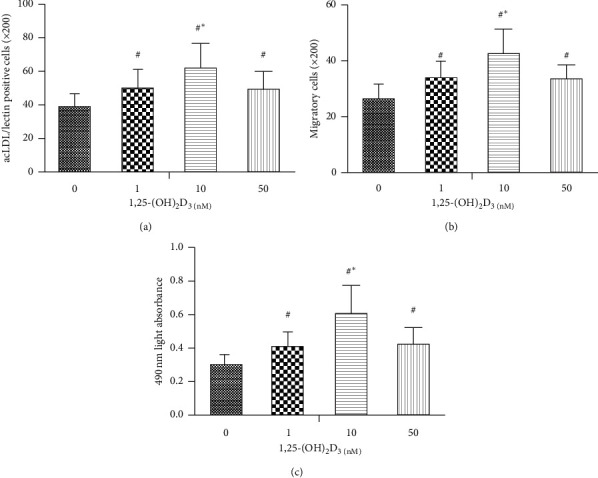
The effect of 1,25-(OH)_2_D_3_ on the number, migration, and proliferation of EPCs *in vitro*. The number, migration, and proliferation of EPCs were significantly increased after culturing in different concentrations of 1,25-(OH)_2_D_3_ (a)–(c). ^#^*P* < 0.05 vs blank control (0 nM 1,25-(OH)_2_D_3_), ^*∗*^*P* < 0.05 vs 1 nM 1,25-(OH)_2_D_3_.

After being cultured *in vitro*, the suspension EPCs from SLE patients were stimulated with different concentrations of 1,25-(OH)_2_D_3_ (0, 1, 10, and 50 nM) for 72 h. The number of EPCs, migration, and proliferation were observed. We found that the number, migration and proliferation of EPCs were all robustly increased after culturing in different concentrations of 1,25-(OH)_2_D_3_ (Figures 3(a)–3(c)).

### 3.3. The Effect of 1,25-(OH)_2_D_3_ on the NO, VEGF, and GM-CSF Secretion by Cultured EPCs

**Figure 4:**
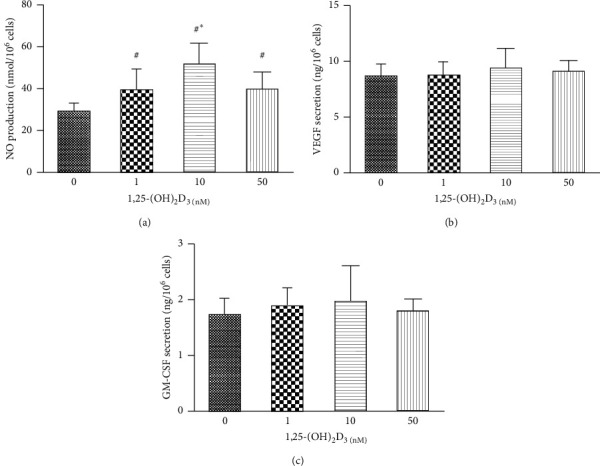
The NO, VEGF, and GM-CSF secretion by EPCs. Different concentrations of 1,25-(OH)_2_D_3_ induced a significant upregulation of NO secretion by cultured EPCs (a), while no difference was observed in VEGF and GM-CSF levels after culturing with 1,25-(OH)_2_D_3_ (b), (c). ^#^*P* < 0.05 vs blank control (0 nM 1,25-(OH)2D3), ^*∗*^*P* < 0.05 vs 1 nM 1,25-(OH)_2_D_3_.

As shown in Figure 4, different concentrations of 1,25-(OH)_2_D_3_ induced a significant upregulation of NO secretion by cultured EPCs (Figure 4(a)), while no difference was observed in VEGF and GM-CSF levels after culturing with 1,25-(OH)_2_D_3_ (Figures 4(b) and 4(c)).

### 3.4. Correlation between NO Level *In Vitro* and the Number and Activity of Circulating EPCs

**Figure 5:**
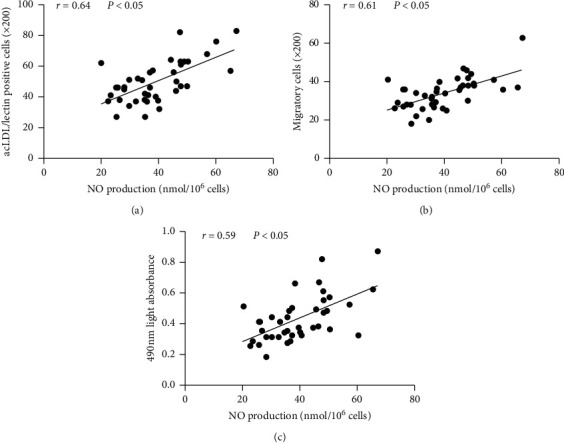
Correlation between the NO level *in vitro* and the number and activity of circulating EPCs. The linear regression relationship between the NO level *in vitro* and promoted circulating EPCs in response to 1,25-(OH)_2_D_3_. There was a significant linear regression relationship between the NO secretion level and the number of cultured EPCs (a). There was a significant linear regression relationship between the NO secretion level and the migratory and proliferative activities of EPCs (b), (c).

There was a significant linear regression relationship between the NO secretion level and the number of cultured EPCs (Figure 5(a)). Similarly, there was a significant linear regression relationship between the NO secretion level and the migratory and proliferative activities of EPCs (Figures 5(b) and 5(c)).

## 4. Discussion

Cardiovascular disease in SLE began with vascular endothelial injury [21]. Recent studies have found that there was a serious deficiency of active vitamin D in SLE, which led to vascular endothelial dysfunction [15, 21]. Our previous clinical observation showed that there was a positive correlation between vitamin D concentration in SLE patients and EPC levels in peripheral blood. However, the effect of 1,25-(OH)_2_D_3_ on the number and function of EPCs in SLE *in vitro* and its mechanism remain unclear.

Several studies have confirmed that vitamin D can improve vascular endothelial function [22, 23]. Pittarella et al. [24] found that 1,25-(OH)_2_D_3_ could directly act on umbilical vein endothelial cells and influence their migration and proliferation through the NO pathway. Martínez-Miguel et al. [25] found that 1,25-(OH)^2D^_3_ can simultaneously upregulate endothelin-1 and NO synthesis in endothelial cells, which play an important role in maintaining the functional balance of endothelial cells.

EPCs in human peripheral blood are a kind of endothelial precursor cells, called circulating EPCs [26]. They are mobilized into peripheral blood by the bone marrow. The cell phenotype is mainly CD34-positive, which can be homing to the site of vascular injury and can differentiate into mature vascular endothelial cells, which can promote angiogenesis in the injured area and repair the injured intima. It has paracrine function and secretes NO, VEGF, and fibroblast factor-2. It also has important repair and protection effects on the endothelial function. Therefore, it plays an important protective role in the occurrence and development of cardiovascular diseases, such as coronary atherosclerotic heart disease and hypertension [8, 14, 27]. In view of the intersection of 1,25-(OH)^2D^_3_ and EPCs in the endothelial function, some scholars began to explore the direct correlation between 1,25-(OH)^2D^_3_ and EPCs.

Cianciolo et al. [28] confirmed that vitamin D receptors also existed in peripheral blood circulation EPCs, and the number of circulating EPCs increased by supplementing active vitamin D to selected dialysis patients. Brodowski et al. [11] found that the endothelial progenitor cell function of preeclampsia pregnant women was improved after vitamin D supplementation. This study further confirmed that there was a positive correlation between vitamin D concentration in SLE patients and the EPCs level in peripheral blood, and 1,25-(OH)^2D^_3_ could directly improve the number, migration, and proliferation of EPCs from human peripheral blood *in vitro*. Therefore, how does 1,25-(OH)^2D^_3_ improve the activity of EPCs?

Vitamin D receptors are divided into nuclear receptors and membrane receptors. They are the main receptors for 1,25-(OH)^2D^_3_ to play biological roles. The vitamin D receptor exists in all kinds of human tissue cells and endothelial cells [30]. Based on the study by Cianciolo et al. [28], we speculated that 1,25-(OH)^2D^_3_ can activate some cell signal transduction pathways by acting on the vitamin D receptors of EPCs, affecting the proliferation, differentiation, and paracrine function of EPCs. Our experiment further examined the function of EPCs to secrete NO. It was found that 1,25-(OH)_2_D_3_ promoted the secretion of NO by EPCs in SLE in a concentration-dependent manner. It was also found that the NO level was closely related to the number, migration, and proliferation of EPCs in SLE *in vitro*. It indicated that the endothelial function of 1,25-(OH)_2_D_3_ in SLE may be achieved by the NO level.

Since the concentration of active vitamin D in the human body is far less than 100 nmol/L [31], we speculated that 1,25-(OH)^2D^_3_ has a limited effect on EPCs *in vivo*. It may only promote the homing of EPCs in peripheral circulation but has little effect on the function of EPCs themselves. More experimental data are needed to further confirm this.

## 5. Conclusion

The present findings indicate that 1,25(OH)^2D^_3_ improves the function of EPCs from SLE patients via NO secretion.

## Figures and Tables

**Table 1:** General clinical characteristics.

Characteristics	SLE (*n* = 20)	Control (*n* = 20)
Age (years)	38.7 ± 12.2	35.1 ± 16.1
BMI (kg/cm^2^)	22.3 ± 3.5	23.5 ± 3.8
Systolic blood pressure (mmHg)	132.8 ± 15.9	130.5 ± 18.4
Diastolic blood pressure (mmHg)	78.4 ± 9.6	76.4 ± 7.4
Heart rate (beats/min)	69.4 ± 8.4	68.5 ± 9.7
AST (mmol/L)	31.4 ± 20.8	38.2 ± 20.7
ALT (mmol/L)	28.2 ± 31.9	33.2 ± 18.7
BUN (mmol/L)	9.0 ± 8.5	7.0 ± 4.8
Cr (mmol/L)	213.7 ± 290.7	185.0 ± 205.1
GLU (mmol/L)	5.9 ± 3.5	5.2 ± 0.8
Serum 25( OH)D (ng/mL)	13.2 ± 6.3^#^	26.6 ± 4.9

Abbreviation: BMI, body mass index; AST, aspartate amino transferals; ALT, alanine transaminase; BUN, blood urea nitrogen; Cr, serum creatinine; GLU: glucose. Notes: Data are given as mean ± SD. ^#^*P* < 0.05 vs control.

## Data Availability

The data used to support the findings of this study are available from the corresponding author or the first author upon request.
